# Efficacy and safety of a booster dose of influenza vaccination in solid organ transplant recipients, TRANSGRIPE 1-2: study protocol for a multicenter, randomized, controlled clinical trial

**DOI:** 10.1186/1745-6215-15-338

**Published:** 2014-08-28

**Authors:** Juliana Martinez-Atienza, Clara Rosso-Fernández, Cristina Roca, Teresa A Aydillo, Joan Gavaldà, Asunción Moreno, Jose M Montejo, Julian Torre-Cisneros, M Carmen Fariñas, Jesus Fortun, Nuria Sabé, Patricia Muñoz, Marino Blanes-Julia, Alejandro Suárez-Benjumea, Francisco López-Medrano, Pilar Pérez-Romero, Elisa Cordero, the TRANSGRIPE 1-2 Study Group

**Affiliations:** Hospital Universitario Virgen del Rocío and Biomedicine Research Institute (IBIS), Infectious Diseases Research Group, Avda. Manuel Siurot, s/n, 41013 Seville, Spain; Hospital Universitario Virgen del Rocío, Clinical Trial Unit, Avda. Manuel Siurot s/n, 41013 Seville, Spain; Hospital Vall d’Hebron, Infectious Diseases Research Group, Passeig de la Vall d’Hebron, 119-129, 08035 Barcelona, Spain; Hospital Clinic, Infectious Diseases Research Group, Carrer Villarroel, 170, 08036 Barcelona, Spain; Hospital Universitario de Cruces, Infectious Diseases Research Group, Plaza de Cruces, 12, 48903 San Vicente de Barakaldo, Vizcaya Spain; Hospital Universitario Reina Sofía, Infectious Diseases Research Group, Avda. Menéndez Pidal, s/n, 14004 Cordoba, Spain; Hospital Universitario Marqués de Valdecilla, Infectious Diseases Research Group, Av Valdecilla, s/n, Santander, 39008 Cantabria, Spain; Hospital Universitario Ramón y Cajal, Ctra. de Colmenar Viejo, km. 9,100, 28034 Madrid, Spain; Hospital Universitario de Bellvitge, Infectious Diseases Research Group, Feixa Llarga, s/n, 08907 L’Hospitalet de Llobregat, Barcelona, Spain; Hospital General Universitario Gregorio Marañón, Infectious Diseases Research Group, Calle Doctor Esquerdo, 46, 28007 Madrid, Spain; Hospital Universitario La Fe, Infectious Diseases Research Group, Avenida Campanar, 21, 46026 Valencia, Spain; Hospital Universitario Virgen Macarena, Infectious Diseases Research Group, Avd. Dr., Fedriani, 341007 Sevilla; Hospital Universitario 12 de Octubre, Infectious Diseases Research Group, Avda de Córdoba, s/n, 28041 Madrid, Spain

**Keywords:** Influenza prevention, Influenza vaccination, Randomized trial, Solid organ transplant

## Abstract

**Background:**

Despite administration of annual influenza vaccination, influenza-associated complications in transplant recipients continue to be an important cause of hospitalization and death. Although influenza vaccination has been proven to be the most effective measure to reduce influenza infection after transplantation, transplant recipients are still vulnerable to influenza infections, with lower serological responses to vaccination compared to the general population. In order to assess the efficacy and safety of an alternative immunization scheme for solid organ transplant recipients, the TRANSGRIPE1-2 Study Group aimed to test a booster dose administration 5 weeks after the standard vaccination. The primary objective of this trial was to compare short-term and long-term neutralizing antibody immunogenicity of a booster dose of influenza vaccination to the standard single-dose immunization scheme. Secondary objectives included the evaluation of the efficacy and/or safety, cellular immune response, incidence of influenza infection, graft rejection, retransplant and mortality rates.

**Methods/Design:**

This phase III, randomized, controlled, open-label clinical trial was conducted between October 2012 and December 2013 in 12 Spanish public referral hospitals. Solid organ transplant recipients (liver, kidney, heart or lung), older than 16 years of age more than 30 days after transplantation were eligible to participate. Patients (*N* = 514) were stratified 1:1 by center, type of organ and time after transplantation and who either received the standard single dose (*n* = 257) or were treated according to a novel influenza vaccination schedule comprising the administration of a booster dose 5 weeks after standard vaccination (*n* = 254). Seroconversion rates were measured as a determinant of protection against influenza (main outcome). Efficacy and safety outcomes were followed until 1 year after influenza vaccination with assessment of short-term (0, 5, 10 and 15 weeks) and long-term (12 months) results. Intention-to-treat, per-protocol and safety analyses will be performed.

**Discussion:**

This trial will increase knowledge about the safety and efficacy of a booster dose of influenza vaccine in solid organ transplant recipients. At the time the manuscript was submitted for publication, trial recruitment was closed with a total of 499 participants included during a 2-month period (within the seasonal influenza vaccination campaign).

**Trial registration:**

ClinicalTrials.gov Identifier: NCT01761435 (registered 13 December 2012).

EudraCT Identifier: 2011-003243-21 (registered 4 July 2011).

## Background

Influenza is a contagious, acute, usually self-limited febrile illness caused by infection with influenza A or B virus that occurs during variable severity outbreaks every winter. Solid organ transplant (SOT) recipients have been reported to be more susceptible to influenza virus [1, 2] compared to the general population. Notwithstanding current vaccination protocols, influenza-associated morbidity and mortality continue to be an important cause of hospitalization and death in SOT recipients [3, 4]. Influenza infection in this group of patients is associated with significant short-term and long-term complications, such as pneumonia, long viral shedding, graft rejection and death [3, 5–9]. Thus, efforts are needed to reduce the incidence of influenza in this especially vulnerable population.

One of the most effective proven measures to reduce the incidence and complications of influenza infection is seasonal influenza vaccination. This strategy has been shown to reduce the risk of graft rejection and death during the first year after transplantation [10]. Although the results of some studies are discordant [11–20], decreased serological responses to influenza vaccination in SOT recipients, compared to the general population, have been reported in most studies.

Despite increasing vaccine coverage throughout the 2010–2011 influenza season, rates of influenza-associated mortality (7.2%) and ICU hospitalization (16.2%) in SOT recipients remained high [21]. Several strategies have been evaluated to improve the efficacy of vaccination in SOT recipients, including the use of higher doses of antigen, administration of adjuvanted vaccines and intradermal route of administration [19, 22–26]. However, these strategies have been assessed only in small cohorts; thus, no clear recommendation can be provided at this time.

Detectable antibody titers against influenza at baseline have been associated with higher postvaccination antibody responses [15, 20]. However, an annual influenza vaccination to maintain baseline antibody titers that may boost yearly vaccine efficacy may not be sufficient in SOT recipients, because only 30% of SOT recipients maintain detectable neutralizing antibody titers 1 year after vaccination [27]. This may be due either to immunosuppressive regimens that, although critical for preventing acute rejection, deplete the adaptive arm of the immune system, or to seasonal vaccine strain modifications that may decrease immunogenicity. A booster dose given some weeks after the standard dose may elicit a long-lasting antibody response, thus producing better protection in current and subsequent influenza seasons. The booster strategy has been tested in several studies involving different types of transplant recipients, but the results are as yet inconclusive [28]. In addition, no randomized controlled trial has addressed this issue to date.

The TRANSGRIPE 1-2 study is a publicly funded, phase III, parallel-group, randomized clinical trial for the evaluation of the safety and efficacy of a booster dose of trivalent inactivated seasonal influenza vaccine administered 5 weeks after seasonal influenza vaccination compared to a single dose of the vaccine administered to SOT recipients. In the present study, we hypothesized that the efficacy of seasonal trivalent inactivated influenza vaccination might be significantly increased in SOT recipients by administering a second booster dose 5 weeks after the standard single-dose vaccination.

The primary objective of the present clinical trial is to compare short-term immunogenicity by measuring neutralizing antibodies after a booster dose versus a standard single dose of influenza vaccine in SOT recipients.

The following are the secondary objectives of the trial:

- To compare the long-term immunogenicity of two doses versus a standard single-dose of influenza vaccine
- To compare short-term and long-term influenza-specific T-cell immune responses among patients within both treatment arms
- To determine the clinical effectiveness of the experimental vaccination scheme, measuring the incidence of confirmed influenza infection
- To establish a safety intervention by analyzing incidence of adverse events (AEs), mortality and outcomes such as graft rejection

Moreover, TRANSGRIPE1-2 trial bears two associated biological substudies in patients who provided specific consent for additional investigations.

## Methods/Design

The TRANSGRIPE1-2 study is a phase III, randomized, controlled, open-label, multicenter clinical trial designed to assess the safety and efficacy of a booster dose of influenza vaccine in SOT outpatients. The study was performed in 12 Spanish referral hospitals between October 2012 and December 2013. The coordinating center was Virgen del Rocío University Hospital, which was responsible for handling clinical trial administrative authorization and regulatory affairs, providing study supplies, independent data and safety monitoring and centralized laboratory immunological quantifications, as well as general coordination and daily operational management of the trial for all of the participating sites. The study was authorized by the Spanish Regulatory Authority and the Coordinating Institutional Review Board of Biomedical Research in Andalusia (Acting as Research Ethics Committee of reference), which gathered the approval from the local ethics committees at all of the participating sites: Virgen del Rocío University Hospital Institutional Review Board (IRB), Seville; Reina Sofía University Hospital IRB, Córdoba; 12 de Octubre University Hospital IRB, Madrid; Gregorio Marañón General University Hospital IRB, Madrid; Ramón y Cajal University Hospital IRB, Madrid; Vall d’Hebron Hospital IRB, Barcelona; Bellvitge University Hospital IRB, Barcelona; Barcelona Clinic Hospital IRB, Barcelona; Cruces University Hospital IRB, San Vicente de Barakaldo; La Fe University Hospital IRB, Valencia; Virgen Macarena University Hospital IRB, Seville; and Marqués de Valdecillas University Hospital IRB, Santander.

### Selection and enrollment

The criteria for patient eligibility are detailed in Table 1. Before the 2012–2013 flu vaccination campaign started, patients were selected from the local SOT recipients lists and invited to participate. Patients who met the selection criteria and agreed to participate in the clinical trial by signing an informed consent form were randomly assigned to the intervention group and subjected to the study protocol, including baseline extraction of blood samples (prior to the administration of standard single-dose influenza vaccination) for the quantification of baseline cellular and humoral immune response.

**Table 1:** **Inclusion and exclusion criteria**

Inclusion criteria	Exclusion criteria
Solid organ transplant recipient (liver, kidney, heart or lung)	Acute graft rejection within 15 days before selection
Age ≥16 years	Documented allergy and/or previous intolerance and/or contraindication to active compounds or excipients or any traces and/or residues in the vaccine
At least 30 days posttransplant	Previous medical record of any severe and/or life-threatening adverse reaction to the vaccine (such as Guillain-Barré syndrome)
Signed informed consent form	Confirmed pregnancy

### Randomization

By 1:1 randomization, patients were allocated either to receive the standard single-dose regimen or to be treated according to a novel influenza vaccination schedule comprising the administration of a booster dose 5 weeks after the first vaccination dose. In order to avoid bias due to differences in immunosuppression level of patients between treatment arms, randomization was stratified according to study sites (because local immunization protocols may differ for SOT recipients), type of organ and time since transplantation. Randomization was centralized and computer-generated, with allocation concealed by integrating a locked list into an electronic case report form (eCRF). A copy of the randomization list is guarded in an opaque, sealed envelope by the trial’s Central Management Team (CMT)at the coordination center that is independent from the investigators who recruited patients.

### Trial intervention and control

Trivalent inactivated influenza vaccine containing World Health Organization–recommended strains for the Northern Hemisphere for the 2012–2013 season was administered. The strains selected for inclusion in the 2012–2013 flu vaccines were an A/California/7/2009(H1N1)pdm09-like virus, an A/Victoria/361/2011(H3N2)-like virus and a B/Wisconsin/1/2010-like virus. The vaccination scheme administered in the two treatment arms is detailed in Table 2: standard intramuscular influenza vaccination (treatment arm A) versus standard vaccination followed by a second booster dose of the same vaccine 5 weeks apart (treatment arm B).

**Table 2:** **Treatment arms**

Treatment arm	Description
Standard influenza vaccination (treatment arm A)	Single intramuscular 0.5-ml dose of inactivated influenza vaccine (split virion) containing World Health Organization (WHO)–recommended strains in Northern Hemisphere and European Union (EU) decision for the 2012–2013 season (A/California/7/2009(H1N1)pdm09-like, A/Victoria/361/2011-like and B/Wisconsin/1/2010-like strains, derived from B/Hubei- Wujiagang/158/2009)
Booster double-dose influenza vaccination (treatment arm B)	Single standard intramuscular 0.5-ml dose of inactivated influenza vaccine (split virion), followed by a second booster dose of the same vaccine 5 weeks later;vaccine strain used was in compliance with the WHO recommendations in Northern Hemisphere and EU decision for the 2012–2013 season (A/California/7/2009(H1N1)pdm09-, A/Victoria/361/2011- and B/Wisconsin/1/2010-like strains, derived from B/Hubei- Wujiagang/158/2009)

The vaccine used for the trial was provided by the Andalusian Health Service, and the pharmacy service of the sponsor’s center was responsible for relabeling and distributing the investigational medicinal product (IMP), according to good manufacturing practices, after obtaining necessary regulatory approval.

Despite the open-label design of the study, all immunologic assays were carried out on coded and anonymous specimens, and data analysts were blinded of which intervention each patient received. During the study period, patients received medical care based on existing local protocols for transplant recipients. Any concomitant medication recommended by clinical practice was allowed within the clinical trial and was registered in the study’s eCRF.

### Follow-up protocol

**Figure 1:**
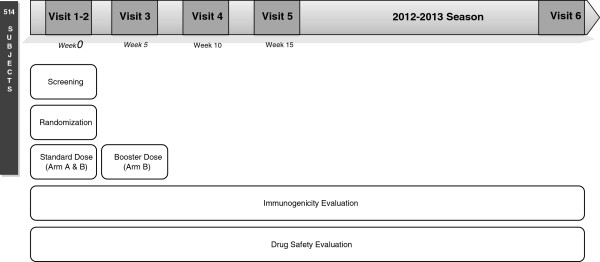
**TRANSGRIPE 1-2 trial design and procedures.** Clinical trial visits are structured in five (or six) time points: initial screening baseline evaluation and administration of the first dose of vaccine in arms A and B (visit 1 and 2), administration in arm B of second dose of vaccine 5 weeks after inclusion (visit 3), short-term follow-up 10 weeks after inclusion (visit 4) and 15 weeks after inclusion (visit 5), as well as long-term follow-up 12 months after the first dose of vaccine (visit 6). Immunogenicity and drug safety were assessed at each programmed time point.

Patients were followed up at the planned visits by clinical teams at the participating centers, ensuring that essential data about efficacy and safety were registered, and samples were collected (Figure 1). The duration of the trial was 14 months: 2 months for patient recruitment and 12 months of follow-up. Clinical, analytical and exploratory data needed for the study were collected and logged in the study’s eCRF at each follow-up visit. For immunogenicity studies, patients’ blood samples were collected at baseline prior to vaccination and at each programmed visit after vaccination. Vaccine efficacy was evaluated at baseline, midterm (5, 10 and 15 weeks after immunization) and long-term (12 months after immunization).

Neutralizing antibody levels for the three influenza strains were measured using microneutralization assays, and seroconversion rates were calculated. Influenza-specific T-cell immune response was measured as the percentage of CD4+ and CD8+ T cells secreting interferon γ in response to influenza stimulation normalized to the negative control.

Clinical trial visits were structured in five (or six) time points as follows:

- *Visits 1 and 2*: Patient screening, baseline evaluation and administration of the first dose of vaccine (arms A and B). Protocols for visits 1 and 2 were carried out in one visit when necessary.
- *Visit 3*: Five weeks after inclusion (range, 25 to 45 days) and administration of the second dose of vaccine (arm B).
- *Visit 4*: Ten weeks after inclusion (range, 60 to 80 days).
- *Visit 5*: Fifteen weeks after inclusion (range, 95 to 115 days).
- *Visit 6*: Twelve months after inclusion (range, 11 to 13 months).

In the programmed visits, patients were prompted to notify the clinician of the onset or continuation of symptoms related to influenza infection occurring during the follow-up period or by email or telephone if symptoms arose during the period between visits. In cases of symptoms, a nasopharyngeal swab was taken for detection of influenza infection using RT-PCR assays.

Each participant had the right to withdraw consent from the study at any point according to international rules. Furthermore, a participant was removed from the study at any time if any of the following withdrawal criteria were met: noncompliance with study procedures and requirements, such as not attending at least two consecutive visits, or if clinical circumstances (for example, AEs, comorbidities) required discontinuation of the study medication or follow-up procedures according to an investigator’s judgment. Patients who left the study fulfilled the study procedure of premature finalization, and AEs were followed until resolution. Statistical analysis was carried out for all the patients who received the first dose of influenza vaccine.

### Study variables

Baseline variables regarding efficacy and safety are described in Table 3 and were measured as appropriate during the follow-up visits.

**Table 3:** **Study variables** ^**a**^

Category	Variables
Demographic data	Sex and age
Comorbidities	COPD, diabetes, obesity, kidney failure, hepatic disease, AIDS, cancer, leukemia, connective tissue disease, cerebrovascular disease, other comorbidities
Previous vaccinations	Influenza vaccination (seasons 2011–2012 and 2010–2011), influenza A H1N1 (2009) vaccination, pneumococcal conjugate vaccination, other immunizations
Solid organ Transplant	Date, organ, immunosuppressive treatment, graft rejection history, use of monoclonal antibodies
Influenza infection records	Confirmed influenza infection in previous season, confirmed influenza infection after study treatment, influenza severity, influenza treatment, influenza treatment outcome, date of recovery
Immune response	Seroconversion and seroprotection rates (GMT, GMR), influenza virus–specific memory A- and B-cell responses, confirmed influenza infection during follow-up
Adverse events	Dates, progress, intensity, expectability, causality, progress, toxicity degree
Concomitant treatments	Indication, dose, frequency, administration route, start and end dates

^a^COPD, Chronic obstructive pulmonary disease; GMR, Geometric mean ratio; GMT, Geometric mean titers.

### Outcome measures

Seroconversion rate was the primary end-point variable of the study. Microneutralization assays were performed to quantify neutralizing antibody titers against the two influenza A strains and the influenza B strain. Immunological response and vaccination efficacy were based on the following international criteria: (1) geometric mean titers (GMT; mean antibody titer in the group of vaccinated individuals), (2) seroprotection rate (percentage of patients with antibody titers ≥1:40), (3) seroconversion rate (percentage of participants with four fold increase in antibody titers from baseline) and (4) geometric mean ratio (GMR; seroconversion factor postvaccination compared with prevaccination). Neutralizing antibody titers were evaluated at 5, 10 and 15 weeks and 1 year after influenza vaccination. Cellular immune response to vaccination was determined by quantifying influenza-specific CD4+ and CD8+ memory cells at baseline, 15 weeks and 1 year after influenza vaccination. For each treatment arm, clinical efficacy was assessed by evaluating the episodes of confirmed influenza infection during a 1-year follow-up period. The safety endpoints at each evaluation time frame were rejection episodes, hospitalization, graft loss, mortality, incidence of AEs and severe AEs and rates of discontinuation due to AEs.

In order to study in-depth the immune response to influenza vaccination in SOT recipients, we will also explore other genetic and biological outcomes in the collected samples. Genetic expression patterns of immune response and anti–human leukocyte antigen levels induced by influenza vaccination will be analyzed as ancillary outcomes.

### Sample size calculation

Sample size was calculated using the results of a Spanish multicenter study in which SOT recipients received a single dose of seasonal influenza vaccination during the 2010–2011 season [27]. In that study, seroconversion rates against influenza A(H1N1) 2009, influenza A(H3N2) and influenza B were 77%, 67% and 67%, respectively. According to these estimates, we calculated our sample size according to the following criteria:

- To achieve a 95% confidence level (significance level of 5%) and 80% statistical power to observe differences in testing the null hypothesis (H0)p1 = p2 using a χ^2^ bilateral test in two independent samples.
- To obtain an increase in the primary endpoint of 10% (increased percentage of seroconversion rate in the experimental arm), which is considered clinically relevant in the literature. According to this criterion, we would expect to achieve 77% and 87% seroconversion rates in the control and experimental arms, respectively.
- To estimate a 10% dropout rate per arm.

According to these premises, and using a 1:1 ratio of experimental units to controls, the minimum number of patients was set at 231 units in each treatment arm, with a total of 462 evaluable participants to be recruited for the study. Taking into account the estimated loss percentage, the total number of patients needed for the study was set at 514 (257 participants per treatment arm). Sample size was calculated using the online Simple Interactive Statistical Analysis calculator [29].

### Statistical analysis

Every effort was made to promote study execution consistency at each participating center with independent monitoring visits to oversee the progress of the trial, ensuring that it was conducted, recorded and reported in accordance with the protocol, standard operating procedures (SOPs), good clinical practice (GCP) and the applicable regulatory requirements.

Both intention-to-treat (ITT) and per-protocol analyses will be performed. The ITT analysis will include all patients who agreed to participate in the study, signed informed consent forms, were randomized and received the first vaccination dose. Per-protocol analysis will include patients who were randomized, received the vaccine according to the assigned scheme (either standard single-dose vaccination in arm A or standard vaccination followed by a booster dose 5 weeks later) and were followed-up, excluding those who discontinued the trial and those for whom severe protocol deviations (violations) were registered. Safety analyses will be performed on the safety population, comprising randomized patients receiving at least the first dose of the influenza vaccine. The safety population is thus equivalent to the ITT population.

The first clinical trial data will be summarized using descriptive statistics. Demographic data at baseline will be analyzed. Other baseline characteristics, including type and date of transplant, basal comorbidities, and immunosuppressive scheme, will be reported for each patient. Summary tables (descriptive statistics and/or frequency tables) will be provided for all baseline variables, efficacy variables and safety variables, as appropriate. A descriptive analysis of continuous variables will be performed (number of patients, mean, standard deviation, range and median). Ninety-five percent confidence highest posterior density intervals may also be presented if appropriate. Frequency counts and percentage of participants within each category will be provided for categorical data.

Efficacy analysis will be performed using seroconversion and seroprotection rates, GMT, GMR and incidence of confirmed influenza infection (to assess clinical effectiveness). The analysis will include, as covariates, previous influenza vaccination, previous record of confirmed influenza infection and other demographic and clinical variables. Additionally, a subgroup analysis will be performed to evaluate effectiveness in patients with decreased response to influenza vaccination (liver failure, use of mammalian target of rapamycin inhibitors, first 6 months posttransplantation).

Vaccination safety analysis will be performed using tabulations of AEs, and descriptive analysis will be provided at baseline and follow-up visits for each treatment group. A tabulation of SAEs will also be provided for each patient within each treatment group. The statistical analysis will be performed by treatment phase (up to 30 days after vaccination) and for the posttreatment phase (long-term follow-up), as appropriate. AEs will be classified on the basis of Medical Dictionary for Regulatory Activities (MedDRA) terminology and summarized for each treatment arm. AE incidence rates will be summarized by system organ class, preferred term, severity and relationship to the IMP. The proportion of patients in each treatment group reporting AEs that occur in ≥3% in each treatment group will be compared using Bayesian methods. The specific system organ classes and preferred terms analyzed will be those reported by at least 5% of the patients in each treatment group.

A bivariate analysis will be performed to compare primary efficacy outcome, secondary efficacy and safety endpoints between treatment arms of the study. A bivariate analysis using a χ^2^ test or Fisher’s exact test will be used for categorical variables, and the Bonferroni correction will be applied when appropriate. For quantitative variables, the Mann–Whitney *U* test or Student’s t-test based on their distribution will be used. If the variances are not homogeneous (Levene test), the Welch test will be applied (analysis of variance). The relative risks and 95% confidence intervals (95% CI) will be calculated by taking the exponent of the natural logarithm of the mean and 95% CI. Also, the linear trend analysis will be used for multiple comparisons. A multivariate model will be used to adjust for possible confounding variables. The threshold of statistical significance will be set at *P* < 0.05. All reported *P*-values will be based on two-tailed tests. We have optimized the design and conduct of the trial to minimize the number of dropouts and missing data. The analysis will be performed on the ITT population, and missing data will be handled by imputing missingness to failure.

All calculations will be performed using SPSS version 18.0 software (SPSS, Chicago, IL, USA). The TRANSGRIPE1-2 clinical trial has not been programmed with any interim analysis or stopping rules, although the incidence of SAEs is being monitored closely to detect a higher frequency of their occurrence, which may require an early termination of the trial.

### Safety and adverse events report

In accordance with GCP, all AEs occurring during the study, as observed by the investigator or reported by the participant, regardless of whether attributed to study medication, were monitored carefully and recorded on the trial eCRFs. The following information was logged: AE description (according to MedDRA terminology), dates of onset and resolution, severity, assessment of causality due to study medication, action taken and other concomitant medications and procedures. Other follow-up information was provided as necessary.

During the course of the clinical trial, investigators reported any SAEs to the clinical monitor within 24 hours of onset. The trial CMT (Central Management Team) was responsible for reporting SAEs to the sponsor, regulatory authorities and ethics committees within the required timelines.

The causality of AEs due to the study medication was assessed by the principal investigator and reevaluated by a qualified person responsible for pharmacovigilance who was appointed by the trial’s CMT (Central Management Team) and independent from the trial’s Scientific Coordination Team (SCT). The study’s pharmacovigilance monitor was responsible for reviewing the accumulated data for participant safety, and efficacy when appropriate, and for making recommendations to the SCT concerning the continuation, modification or termination of the trial.

AEs related to the study medication, as judged by the pharmacovigilance monitor, were followed until resolution or until stable. All related AEs that resulted in a participant’s withdrawal from the study or were present at the end of the study will be followed until satisfactory resolution. The decision whether an AE was of sufficient severity to require participant removal from treatment was left to the investigator’s clinical judgment. If this happened, according to GCP rules, the participant was asked to attend to an end-of-study assessment visit and was given appropriate care under medical supervision until symptoms ceased or became stable.

### Project management

The trial is supported by the Clinical Trial Unit at Virgen del Rocío University Hospital and was carried out in accordancewith all relevant SOPs for the conduct, management and monitoring of the study.

Strategic management of the trial was the responsibility of the SCT, comprising the trial coordinating investigator and the local research staff. The SCT was responsible for supervising trial enrollment and patient follow-up and offering practical clinical perspectives to assist study local teams with medical aspects of the study implementation. The SCT was also in charge of data management and statistical analysis.

The Biomedicine Research Institute of Seville at the coordinating trial site provided qualified research staff and access to laboratory facilities to assist in sample logistics, processing of samples, carrying out experimental procedures and biological research and providing expertise.

Operational management of the study was the responsibility of the CMT, comprising the TRANSGRIPE1-2 project manager, the clinical monitor, the person responsible for pharmacovigilance, and the assisting research staff at the clinical trial unit of the coordinating trial site. The CMT was responsible for handling clinical trial administrative authorization and regulatory affairs, day-to-day trial operations management and independent data and safety monitoring. The CMT also took responsibility for drug safety and pharmacovigilance tasks. The IMP sourcing and distribution were managed by a qualified pharmacist in the coordinating site’s pharmacy service under the supervision of the CMT.

The STC and CMT worked independently, meeting at least quarterly for overall supervision of the progress of the clinical trial, guaranteeing adherence to the planned timescale, and ensuring that it was conducted, recorded and reported in accordance with the protocol, SOPs, GCPs and applicable local regulatory requirements.

### Data and safety monitoring

The aim of monitoring is to ensure patient protection, data quality and trial integrity. To ensure that investigators were following the protocol, complying with regulatory and GCP standards and collecting and reporting quality data, a clinical monitor or clinical research associate appointed by the trial’s CMT was responsible for supervising study progress at each investigational site throughout the duration of the study. Monitoring involved periodic on-site visits, as well as centralized supervision activities to identify, prevent or mitigate risks regarding data quality and for patient protection and trial integrity.

### Ethical, deontological and regulatory considerations

Investigators ensured that this study was conducted in accordance with the principles of the Declaration of Helsinkiand the International Conference Harmonization(ICH) Guidelines for GCP and in full conformity with applicable regulations.

The protocol, informed consent form, participant information sheets and any applicable documents received full written ethical and regulatory authority approval. The trial is registered in publicly accessible databases such as the EU Clinical Trials Register and ClinicalTrials.gov. All substantial amendments to the original approved documents also received further approval from the corresponding ethics committee and regulatory authority.

Once patients met the trial selection criteria, and prior to study enrollment, investigators obtained their written informed consent after providing them with appropriate information about the effects, objectives, methods, anticipated benefits and potential risks of participation. Investigators also explained the right to withdraw consent at any time for any reason. If a patient was unable to read or provide consent, the patient’s legal representative was present during the informed consent process and signed the consent form.

The trial staff ensured that participants’ confidentiality was preserved. Trial participants were identified by a code on the eCRF. All study documents and data were stored securely and were accessible only by the principal investigator and the authorized staff for purposes related tothe trial. Individual user identifications and passwords were necessary to access the trial eCRFs. eCRF data were encrypted into an SPSS database and stored under the custody of the trial’s CMT.

The CONSORT guidelines [30] are guaranteed when publishing the study results in clinical journals and presenting them at national and international conferences.

## Discussion

Despite annual vaccination protocols, influenza-associated complications continue to be an important cause of hospitalization and death in SOT recipients [3, 4]. Although influenza vaccination has proven to be the most effective measure to reduce influenza infection after transplantation [10], SOT recipients are still vulnerable to influenza infections because their immunological response to vaccination is lower than that of the general population. Thus, efforts are needed to improve influenza vaccine immunogenicity, especially in this vulnerable population with immunosuppression.

Previous researchers have reported a booster effect of seasonal influenza vaccine in patients who have baseline antibody titers [15, 20]. In a prospective multicenter study, investigators observed that patients with baseline titers had significantly higher sero conversion rates than patients with no baseline antibody titers (90.9% versus 73.0% respectively, for the 2009H1N1 strain and 92.2% versus 62.2%, respectively, for the H3N2 strain). Moreover, patients without preexisting seroprotection at baseline had lower antibody titers after influenza vaccination, whereas patients with baseline antibody titers reached higher GMTs after vaccination [27]. The hypothesis of the present trial is based on these premises, and we aimed to evaluate the safety and efficacy of a second, booster dose of influenza vaccine administered 5 weeks after the standard seasonal influenza vaccination in SOT recipients.

Preliminary results based on evaluation of the efficacy of a booster dose of influenza vaccination in SOT recipients are controversial. Soesman *et al*. [17] observed an increased serological rate in liver transplant recipients after the second dose of influenza vaccine (80% double-dose versus 67% single-dose), whereas other studies have shown improved efficacy only after a third dose [18]. Conversely, other authors have found no benefits of administering a second vaccine dose [15, 31]. Nevertheless, available evidence is scarce, primarily owing to small sample sizes of the studies, high baseline seroprotection rates, lack of randomization and conditions that are not applicable to the present clinical context, such as different immunosuppression schemes or different circulating virus serotypes [20, 31].

These issues encourage identification of solid base of research evidence, which is necessary for implementation of novel strategies to improve influenza prevention in transplant recipients. Therefore, in the present phase III clinical trial, we intended to evaluate prospectively the safety and efficacy of a booster dose of influenza vaccine versus standard single-dose vaccination in parallel cohorts of SOT recipients.

## Conclusions

The solid scientific basis of our hypothesis, the adequate design of the study and the efforts invested to meet regulatory requirements and international standards for clinical trials will be able to produce solid evidence about the use of a booster dose of influenza vaccination in SOT recipients. Furthermore, these findings will contribute to development and implementation of strategies to improve influenza vaccination response, especially early after transplantation, where the risk of severe complications due to influenza infection is higher.

## Trial status

At the time this manuscript was submitted the TRANSGRIPE1-2 trial was closed. The recruitment period was closed in December 2012 with a total of 499 participants enrolled, and follow-up visits were completed in December 2013.

## Abbreviations

AE: Adverse event
CI: Confidence interval
CMT: Central Management Team
COPD: Chronic obstructive pulmonary disease
eCRF: Electronic Case Report Form
GMR: Geometric mean ratio
GMT: Geometric mean titers
ICH: International Conference Harmonization
IMP: Investigational medicinal product
ITT: Intention-to-treat
PI: Principal investigator
SAE: Serious adverse event
SCT: Scientific Coordination Team
SOP: Standard operating procedure
SOT: Solid organ transplant.
